# Effects of Blood Flow Restriction Training on Muscle Strength and Hypertrophy in Untrained Males: A Systematic Review and Meta-Analysis Based on a Comparison with High-Load Resistance Training

**DOI:** 10.3390/life14111442

**Published:** 2024-11-07

**Authors:** Hualong Chang, Jie Zhang, Jing Yan, Xudong Yang, Biao Chen, Jianli Zhang

**Affiliations:** 1 College of Physical Education and Health Sciences, Zhejiang Normal University, Jinhua 321004, China; 19558257203@163.com (H.C.);; 2 College of Education, Anyang Normal University, Anyang 455000, China; 3 Department of Sports Science, Chungnam National University, Daejeon 34134, Republic of Korea; 4 Renji College, Wenzhou Medical University, Wenzhou 325035, China; 5 Exercise and Metabolism Research Center, College of Physical Education and Health Sciences, Zhejiang Normal University, Jinhua 321004, China

**Keywords:** KAATSU training, low-load resistance, high-load resistance, muscle strength, male

## Abstract

This meta-analysis examined the efficacy of low-load resistance training with blood flow restriction (LL-BFR) versus high-load resistance training (HL-RT) on muscle strength and hypertrophy, exploring factors affecting outcomes. We searched Embase, CNKI, Wanfang, PubMed, Ovid Medline, ProQuest, Cochrane Library, Embase, and Scopus from inception to July 2024. After assessing the risk of bias using the Cochrane tool, a meta-analysis was conducted to calculate the overall effect size. Subgroup analyses were performed to explore the impact of different modulating factors on training effects. LL-BFR was found to be inferior to HL-RT with regard to muscle strength gains (SMD = −0.33, 95% CI: −0.49 to −0.18, *p* < 0.0001). However, subgroup analyses revealed that LL-BFR achieved muscle strength gains comparable to HL-RT under individualized pressure (SMD = −0.07, *p* = 0.56), intermittent cuff inflation (SMD = −0.07, *p* = 0.65), and a higher number of training sessions (SMD = −0.12, *p* = 0.30). No significant difference in muscle mass gains was observed between LL-BFR and HL-RT (SMD = 0.01, *p* = 0.94), and this conclusion remained consistent after controlling for modulating variables. HL-RT is superior to LL-BFR in enhancing muscle strength gains. Nevertheless, under appropriate conditions, including individualized pressure prescription, intermittent cuff inflation, and a higher number of training sessions, LL-BFR can achieve muscle strength gains comparable to HL-RT, emphasizing the importance of tailored training programs. Both methods exhibit similar effects on muscle mass gains, indicating that LL-BFR serves as an effective alternative for individuals who cannot perform HL-RT because of physical limitations or injury concerns.

## 1. Introduction

Muscular strength and mass play pivotal roles in human health [1] and athletic performance [2]. A robust muscular system not only supports the body, enhances joint and bone stability, and reduces the risk of potential injuries during daily activities [3], but also plays a crucial role in regulating metabolism, promoting efficient energy expenditure, and maintaining a healthy weight and body composition [4,5]. For a long time, high-load resistance training (HL-RT, 65–85% one-repetition maximum [1RM]) has been considered the traditional gold standard for enhancing muscular strength and mass [6,7]. HL-RT stimulates muscle growth and strength improvement by exerting sufficient resistance to promote high-intensity muscle contractions [8]. However, for beginners, the elderly, and individuals with chronic diseases or unhealed injuries, HL-RT may pose a higher risk of injury or physical burden.

With this background, low-load resistance training (LL-RT), particularly when combined with blood flow restriction (low-load blood flow restriction [LL-BFR]), also known as blood flow restriction training (BFRT), has received considerable attention because of its lower entry threshold. By applying external pressure to the proximal limbs during exercise, LL-BFR achieves gains in muscular strength and mass under low-load conditions. The core mechanism lies in the hypoxic state induced by pressure, forcing compensatory adaptation in muscles, thereby triggering muscle growth [9]. Compared with LL-RT alone, LL-BFR has been proven to yield superior results in muscular strength and mass gains [10,11].

Some studies indicate that LL-BFR can achieve comparable training effects to HL-RT [12,13], while others suggest that LL-BFR may be slightly inferior to HL-RT with regard to muscular strength training [14,15,16]. Previous meta-analyses have shown that HL-RT outperforms LL-BFR in muscular strength training, even after controlling for certain moderators, but the differences in muscle mass gains are not significant [17]. Another meta-analysis suggests that individualized pressure and releasing cuff pressure during rest periods in LL-BFR achieves comparable muscular strength gains to HL-RT [18], and the meta-analysis does not delve into the specific effects on muscle mass. This suggests that the method of cuff pressure application may influence the training effectiveness of LL-BFR. Additionally, there is a lack of research exploring the impact of cuff pressure application methods on muscle strength and hypertrophy in untrained men.

Therefore, this study focuses on untrained male individuals and aims to objectively assess the specific effects of LL-BFR and HL-RT on their muscular strength and mass through scientific methods. The study uses subgroup analysis to delve into the specific mechanisms underlying the influence of various pressure application characteristics (such as intermittent versus continuous pressure application and individualized pressure settings) and other unexplored moderating factors on the training outcomes.

## 2. Materials and Methods

This study adhered strictly to the most recent guidelines set forth by the Preferred Reporting Items for Systematic Reviews and Meta-Analyses (PRISMA). Additionally, it has been officially registered with PROSPERO, bearing the registration number CRD42024561007.

### 2.1. Search Strategy

Two independent researchers systematically searched multiple electronic databases, including CNKI, Wanfang, PubMed, Ovid Medline, ProQuest, Cochrane Library, Embase, and Scopus, spanning from the inception of each database until July 2024. The articles were searched using the following specific keywords: “blood flow restriction training” or “occlusion training” or “vascular occlusion” or “KAATSU” and or “muscle strength” or “muscle mass” or “hypertrophy” and “randomized controlled trial” or “RCT”. Furthermore, both researchers conducted a manual search of the reference lists of the retrieved articles to uncover any potentially eligible articles that, despite possibly being overlooked, fulfilled the study criteria. The comprehensive search strategy employed in the PubMed database is outlined in Supplementary Table S1.

### 2.2. Inclusion and Exclusion Criteria

**Figure 1:**
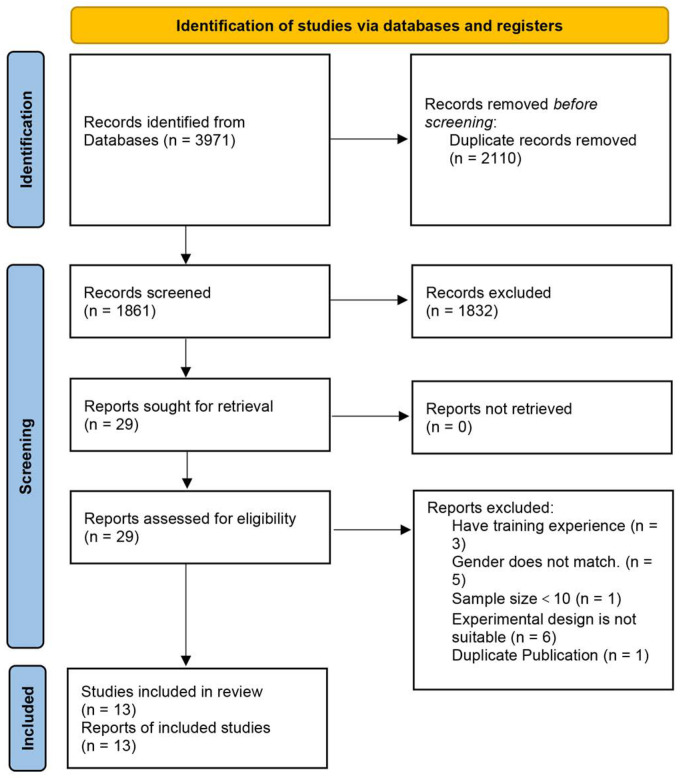
Flowchart illustrating the study selection process based on the latest PRISMA guidelines.

Stringent inclusion and exclusion criteria were established on the basis of the PICOS framework. The inclusion criteria were as follows: (1) exclusively healthy adults aged 18–60 years (participants); (2) undergoing non-vascular occlusion resistance training with a load ≥65% 1RM (control group); (3) undergoing vascular occlusion resistance training with a load <50% 1RM (intervention group); (4) changes in muscle strength assessed via dynamic, isometric, or isokinetic testing, or muscle hypertrophy evaluated through ultrasound or magnetic resonance imaging with regard to the cross-sectional area, muscle mass, and thickness, measured both pre- and post-intervention (outcome measures); (5) RCTs that clearly delineate the type of training—whether hypertrophy- or strength-focused—in both the control and intervention arms (study type). The exclusion criteria were established as follows: (1) studies involving animals; (2) research focusing on the impact of dietary supplements on muscle strength; (3) designs in which the experimental and control groups were composed of the left and right sides of the same individual; (4) non-original research articles, including protocols, meta-analyses, or systematic reviews; and (5) articles not published in Chinese or English. Both researchers independently scrutinized and assessed each article against the inclusion and exclusion criteria. In cases of disagreement, they resolved the discrepancies through discussions with a third researcher to guarantee consistency and rationality in the final selections. The detailed process of study selection, adhering to the PRISMA guidelines, is illustrated in Figure 1.

### 2.3. Outcome Measures and Data Extraction

Using Excel spreadsheets, two researchers independently extracted key characteristics from each article, including (1) the first author’s surname; (2) participant characteristics (number and gender); (3) exercise/intervention specifics (exercise load, duration, and frequency); (4) types of muscle strength tests and hypertrophy assessment metrics with muscle groups; and (5) percentage increases in outcome measures between the experimental and control groups. For data presented graphically, the Web Plot Digitizer tool was employed. The percentage changes in muscle strength and mass were calculated ([((Meanpost − Meanpre)/Meanpre) × 100]) and reported as the minimum and maximum averages across various assessment methods (Tables S2 and S3). Notably, in multi-period interventions, the most recent available time point was analyzed for muscle strength assessment. The research team cross-checked and analyzed the extracted data thoroughly, contacting corresponding authors when necessary for missing or additional data to ensure accuracy.

### 2.4. Quality Assessment for the Included Trials

The Cochrane Collaboration’s tool for assessing the risk of bias was employed [19], covering areas such as random sequence generation and allocation concealment (assessing selection bias), blinding of participants/personnel (assessing performance bias), blinding of outcome assessment (assessing detection bias), completeness of outcome data (assessing attrition bias), selective reporting (assessing reporting bias), and other biases. Two researchers independently conducted the bias risk assessment, resolving disagreements through discussion and involving a third researcher if necessary.

### 2.5. Statistical Analysis

To synthesize the effects of LL-BFR interventions on muscle strength and hypertrophy in untrained healthy adults, statistical analysis was performed using RevMan 5.4. Depending on heterogeneity, a fixed-effect model was applied to calculate standardized mean differences (SMDs) with 95% confidence intervals (95% CI). Heterogeneity among the included studies was assessed using the I^2^ statistic, with <25% indicating low heterogeneity, 25–75% indicating moderate heterogeneity, and >75% indicating high heterogeneity. Pooled effect sizes (ES) were calculated for each comparison, with statistical significance set at *p* < 0.05. Data were reported as mean ± standard deviation.

This study first compared the effectiveness of LL-BFR in enhancing muscle strength and mass with that of HL-RT. Subsequent subgroup analyses delved into the specific impacts of different occlusion pressure prescriptions (individualized vs. non-individualized), cuff inflation modes (continuous vs. intermittent pressure), and numbers of training sessions on muscle strength and mass gains.

Sensitivity analyses were conducted to assess the robustness of the meta-analysis results by sequentially excluding each study and rerunning the analysis. If the estimated value after excluding a study fell outside the 95% CI of the combined effect, then that study was deemed to have a significant influence on the combined result, which may indicate potential bias. Finally, funnel plots were constructed, and Egger’s test was performed using Stata version 12 to detect publication bias.

## 3. Results

### 3.1. Search Results and Selection of Studies

The systematic literature search yielded a total of 3971 articles, encompassing English-language databases such as PubMed (521 articles), Ovid Medline (457 articles), ProQuest (266 articles), Cochrane Library (959 articles), Embase (491 articles), and Scopus (702 articles), along with Chinese-language databases such as CNKI (142 articles) and Wanfang Data (433 articles). After eliminating 2110 duplicate articles, 1861 were included in the initial evaluation. Subsequently, the titles and abstracts of these 1861 articles were scrutinized, resulting in the exclusion of 1832 articles that did not meet the eligibility criteria based on the research objectives and inclusion standards. Further in-depth full-text examination of the remaining 29 articles confirmed their suitability for qualitative analysis. Ultimately, 13 articles were included in the qualitative analysis. Figure 1 illustrates the entire process of literature search and screening in a flowchart format.

### 3.2. Summary of the Included Studies

#### 3.2.1. Study Characteristics and Participants

Based on the inclusion and exclusion criteria, 13 articles published between 2011 and 2023 were selected for analysis [10,12,13,14,15,16,20,21,22,23,24,25,26]. The study participants were adult males with no training experience or who had not engaged in systematic resistance training within the past 2 months. A total of 336 subjects were included, with 147 in the LL-BFR group and 189 in the HL-RT group. The age range was 18–45 years, with an average age of approximately 25 years.

#### 3.2.2. Intervention Characteristics

In the LL-BFR group, interventions involved low loads of 20–40% of 1RM combined with blood flow restriction. On the contrary, the HL-RT group utilized high loads ranging from 70% to 90% of 1RM. The training frequency varied among studies, with twelve articles reporting 2 to 3 days per week and one article reporting 4 days per week. The training duration typically lasted 4 to 12 weeks.

When assessing the effects of LL-BFR and HL-RT on muscle strength, eight articles indicated comparable gains between the two methods, four articles reported lower strength gains with LL-BFR compared with HL-RT, and one article suggested greater strength gains with LL-BFR. Regarding muscle mass, all thirteen articles concurred that LL-BFR and HL-RT produced equivalent increases, with one article noting that lower loads in LL-BFR resulted in less muscle mass gain compared with HL-RT. With regard to occlusion pressure prescription, six articles employed individualized pressure, whereas seven used non-individualized pressure. For cuff inflation modes, four articles utilized intermittent pressure and ten used continuous pressure. Tables S2 and S3 provide detailed data on demographic and exercise characteristics.

### 3.3. Summary of Risk of Bias

**Figure 2:**
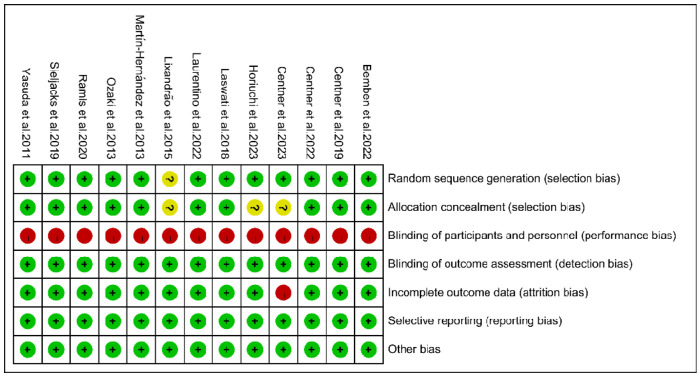
Risk of bias summary plot of included studies [10,12,13,14,15,16,20,21,22,23,24,25,26].

**Figure 3:**
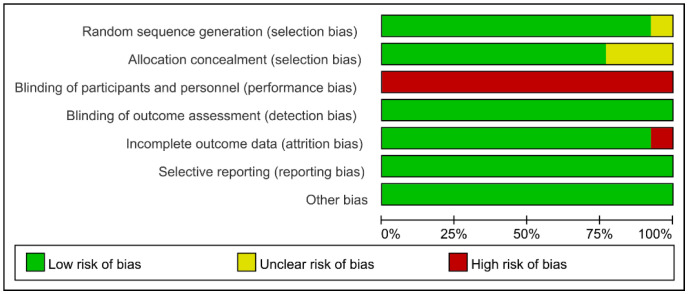
Summary of literature quality assessment.

As depicted in Figure 2 and Figure 3, green, yellow, and red represent low, unclear, and high risks, respectively. With regard to selection bias, twelve articles demonstrated low risk for random sequence generation, but three did not report allocation concealment. Given that LL-BFR was the primary intervention in all trials, blinding of participants and researchers may not have been feasible, leading to a high risk of performance bias. However, this limitation did not significantly affect the reliability of data analysis or conclusions. No studies exhibited risk of measurement bias; only one study had a risk of attrition bias, and no studies had a reporting bias or other biases. Studies were considered to have an overall low risk of bias if all items were rated as “low risk”, a moderate risk if 1–2 items were rated as “high risk” or “unclear”, and a high risk if more than two items received such ratings. In summary, eleven studies were rated as having an overall moderate risk of bias, and two studies were rated as having an overall high risk of bias.

### 3.4. Results of the Meta-Analysis

This section reports the findings of the meta-analysis on muscle strength and mass gains, where different letters within the same study are used to distinguish distinct outcome assessment methods (Figure 4, Figure 5, Figure 6, Figure 7, Figure 8, Figure 9, Figure 10 and Figure 11).

#### 3.4.1. Result of Muscle Strength

**Figure 4:**
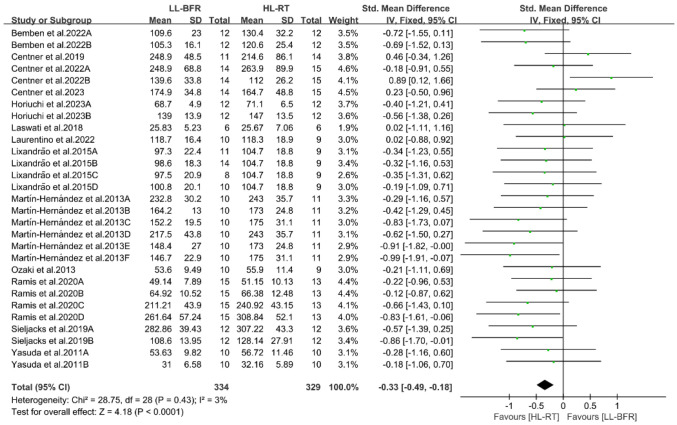
Effects of LL-BFR and HL-RT on muscle strength [10,12,13,14,15,16,20,21,22,23,24,25,26].

A meta-analysis was performed on 13 articles, encompassing 29 study outcomes, to assess and compare the variations in muscle strength gains between the LL-BFR and HL-RT groups. The pooled SMD indicated extremely low heterogeneity (I^2^ = 3%, *p* = 0.43). Thus, a fixed-effect model was used for analysis. The summary results of the meta-analysis (Figure 4) reveal that the HL-RT group achieved significantly greater muscle strength gains than the LL-BFR group (SMD = −0.33, 95% CI: −0.49 to −0.18, *p* < 0.0001).

**Figure 5:**
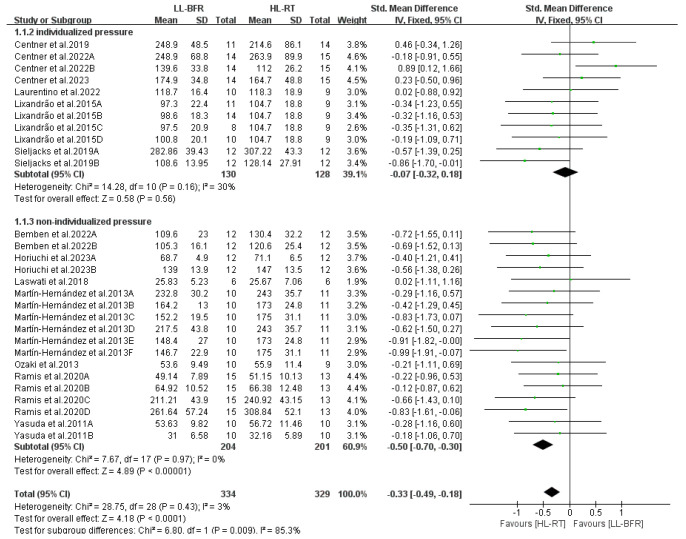
Effects of occlusive pressure prescription on muscle strength [10,12,13,14,15,16,20,21,22,23,24,25,26].

Further analysis explored the potential moderating effects. Figure 5 presents the subgroup analysis results regarding the occlusion pressure prescription in LL-BFR. The results indicate that when individualized pressure was applied, muscle strength gains in the LL-BFR group were comparable to those in the HL-RT group (SMD = −0.07, 95% CI: −0.32 to 0.18, *p* = 0.56, I^2^ = 30%); however, when non-individualized pressure was used, muscle strength gains in the LL-BFR group were lower than those in the HL-RT group (SMD = −0.50, 95% CI: −0.70 to −0.30, *p* < 0.00001, I^2^ = 0%).

**Figure 6:**
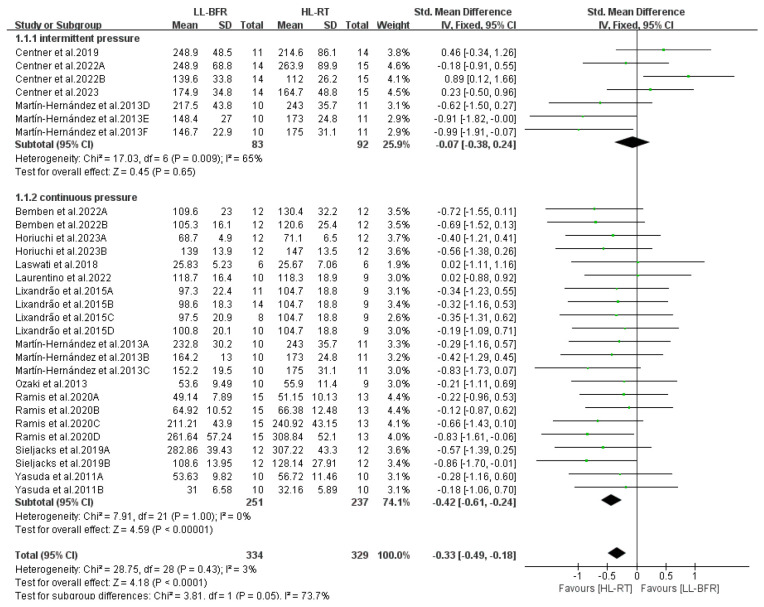
Effects of cuff inflation patterns on muscle strength [10,12,13,14,15,16,20,21,22,23,24,25,26].

Figure 6 displays the subgroup analysis results for cuff inflation modes in LL-BFR. When cuff pressure was released during inter-set rests (i.e., intermittent pressure), muscle strength gains in the LL-BFR group were comparable to those in the HL-RT group (SMD = −0.07, 95% CI: −0.38 to 0.24, *p* = 0.65, I^2^ = 65%). By contrast, when cuff pressure was maintained throughout the training session (i.e., continuous pressure), muscle strength gains in the LL-BFR group were lower than those in the HL-RT group (SMD = −0.42, 95% CI: −0.61 to −0.24, *p* < 0.00001, I^2^ = 0%).

**Figure 7:**
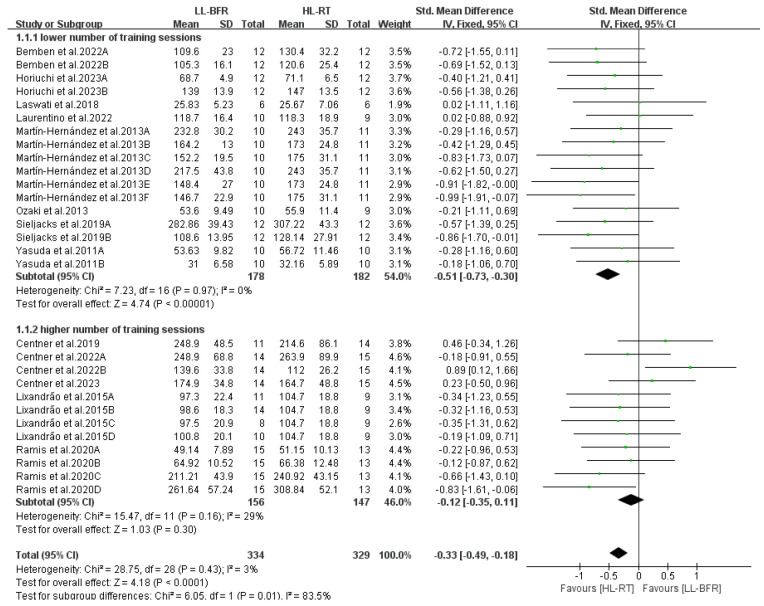
Effect of number of training sessions on muscle strength [10,12,13,14,15,16,20,21,22,23,24,25,26].

Figure 7 presents the subgroup analysis results regarding the number of training sessions undertaken by participants. The results indicate that when there was a lower number of training sessions (*n* ≤ 18), the muscle strength gains in the LL-BFR group were lower than those in the HL-RT group (SMD = −0.51, 95% CI: −0.73 to −0.30, *p* < 0.00001, I^2^ = 0%); however, when there was a higher number of training sessions (*n* > 18), muscle strength gains in the LL-BFR group were comparable to those in the HL-RT group (SMD = −0.12, 95% CI: −0.35 to 0.11, *p* = 0.30, I^2^ = 29%)

#### 3.4.2. Result of Muscle Mass

**Figure 8:**
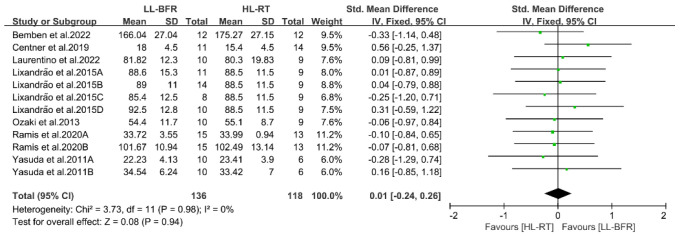
Effects of LL-BFR and HL-RT on muscle mass [10,12,13,14,20,22,23].

Seven randomized controlled trials (RCTs), encompassing a total of 12 study outcomes and meeting the inclusion criteria, were analyzed to assess and compare the variations in muscle mass gains between the LL-BFR and HL-RT groups. Given the extremely low heterogeneity among studies (I^2^ = 0%, *p* = 0.98), a fixed-effect model was employed for comprehensive analysis of the differences in muscle mass gains between the LL-BFR and HL-RT groups. As shown in Figure 8, the meta-analysis results indicate no significant difference in muscle mass gains between the LL-BFR and HL-RT groups (SMD = 0.01, 95% CI: −0.24 to 0.26, *p* = 0.94), indicating that both training methods can enhance muscle mass.

**Figure 9:**
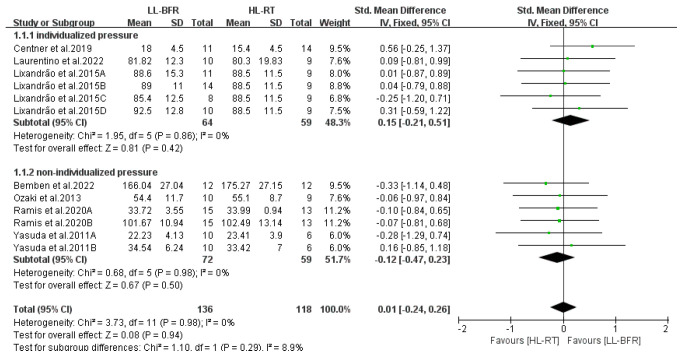
Effect of occlusive pressure prescription on muscle mass [10,12,13,14,20,22,23].

To further refine the results, subgroup analyses were conducted, but no significant moderating factors were identified. As depicted in Figure 9, when individualized pressure was applied, muscle mass gains in the LL-BFR group were comparable to those in the HL-RT group (SMD = 0.15, 95% CI: −0.21 to 0.51, *p* = 0.42, I^2^ = 0%). Similarly, when non-individualized pressure was used, muscle mass gains in the LL-BFR group were also comparable to those in the HL-RT group (SMD = −0.12, 95% CI: −0.47 to 0.23, *p* = 0.5, I^2^ = 0%).

**Figure 10:**
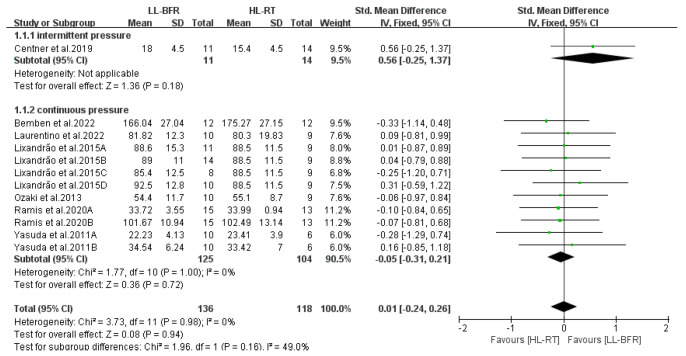
Effect of cuff inflation pattern on muscle mass [10,12,13,14,20,22,23].

Figure 10 displays the subgroup analysis results examining the impact of cuff inflation modes on muscle mass changes in LL-BFR. In this study, when intermittent pressure was applied during inter-set rests, muscle mass gains in the LL-BFR group were comparable to those in the HL-RT group (SMD = 0.56, 95% CI: −0.25 to 1.37, *p* = 0.18, I^2^ not applicable). Similarly, when continuous pressure was applied throughout the training session, muscle mass gains in the LL-BFR group were equivalent to those in the HL-RT group (SMD = −0.05, 95% CI: −0.31 to 0.21, *p* = 0.72, I^2^ = 0%).

**Figure 11:**
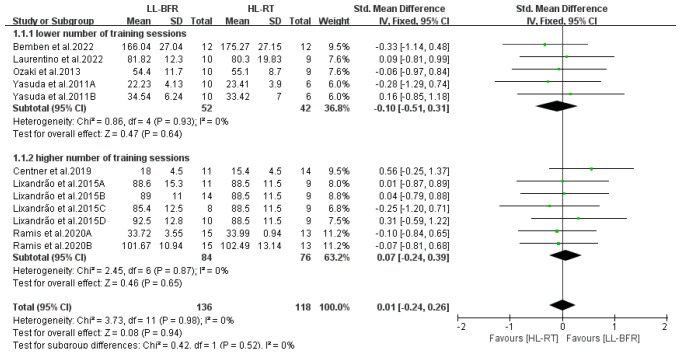
Effect of number of training sessions on muscle mass [10,12,13,14,20,22,23].

Figure 11 presents the subgroup analysis results regarding the number of training sessions undertaken by participants. The results indicate that when there was a lower number of training sessions (*n* ≤ 21), muscle mass gains in the LL-BFR group were comparable to those in the HL-RT group (SMD = −0.10, 95% CI: −0.51 to 0.31, *p* = 0.64, I^2^ = 0%). Furthermore, when there was a higher number of training sessions (*n* > 21), muscle mass gains in the LL-BFR group remained equivalent to those in the HL-RT group (SMD = −0.07, 95% CI: −0.24 to 0.39, *p* = 0.65, I^2^ = 0%).

#### 3.4.3. Sensitivity Analysis

**Figure 12:**
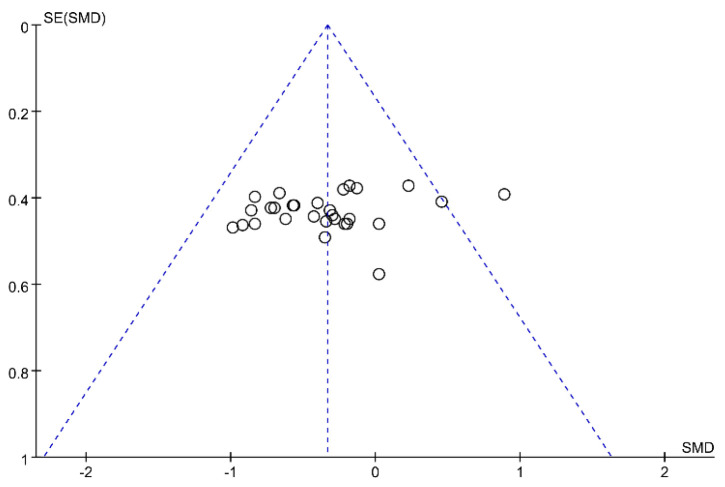
The muscle strength publication bias funnel plot. The circles represent study outcomes.

Sensitivity analysis revealed that the exclusion of any single study did not significantly alter the overall findings when assessing muscle strength gains, indicating the robustness of the meta-analysis results and their independence from any individual study. In addition, funnel plot analysis (Figure 12) demonstrated that although one study fell outside the boundary, it did not constitute significant publication bias, thereby indicating a slight deviation. This finding was further corroborated by Egger regression analysis (*p* = 0.226).

**Figure 13:**
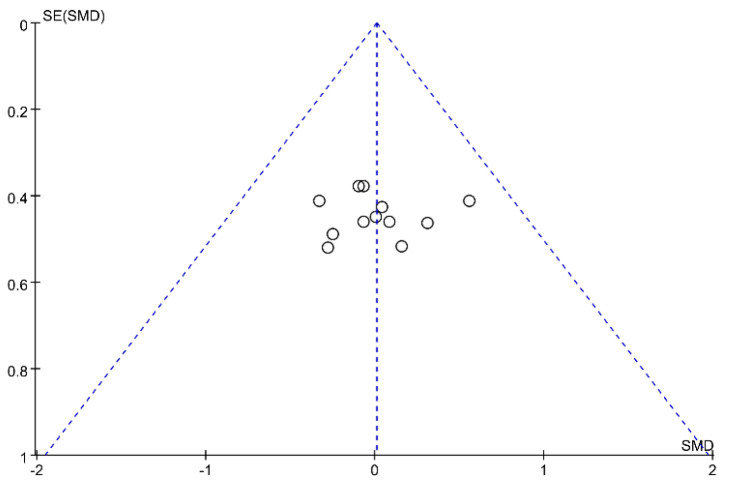
The muscle mass publication bias funnel plot. The circles represent study outcomes.

Similarly, in the evaluation of muscle mass gains, the sensitivity analysis failed to identify any notable impact of individual studies on the results. The funnel plot analysis (Figure 13) and the Egger regression analysis (*p* = 0.932) indicated the absence of significant publication bias.

## 4. Discussion

In this study, multiple experimental data and cases were comprehensively analyzed to systematically explore the effects of LL-RT combined with blood flow restriction (LL-BFR) and HL-RT on enhancing muscle strength and hypertrophy among untrained males. The results indicate that HL-RT exhibits a more pronounced advantage in muscle strength gains, whereas LL-BFR remains effective in increasing muscle mass at lower loads. This finding holds remarkable implications for athletes’ rehabilitation programs and beginners’ daily fitness routines. Despite low heterogeneity among studies, a deeper examination of potential moderating factors is crucial to understand the differences in the effects of LL-BFR and HL-RT, particularly occlusion pressure prescriptions, cuff inflation modes, and the number of training sessions in LL-BFR. Subgroup analyses revealed that individualized pressures, intermittent pressure application patterns, and a high number of training sessions remarkably improved muscle strength gains in the LL-BFR group, thereby achieving comparable growth levels to the HL-RT group under certain conditions. This discovery provides a solid basis for personalizing LL-BFR training protocols. Previous research has focused on the influence of training frequency and duration, but this study underscores the potential accuracy of subgrouping based on the number of training sessions. The clustering approach adopted in this study, which divides subgroups based on the median training sessions across included studies, provides a novel perspective and methodology for research in this field. Similar to muscle strength, moderation effects were tested for muscle mass, but individualized pressure, cuff inflation mode, and training sessions showed no significant influence on muscle mass. This result further supports our conclusion that LL-BFR and HL-RT are comparable in enhancing muscle mass regardless of these moderating factors. To our knowledge, this meta-analysis is the first to focus on the effects of BFRT on muscle strength and hypertrophy in untrained male adults, who were untrained or had no resistance training experience for at least 2 months, ensuring the specificity and reliability of our findings.

### 4.1. Muscle Strength

The findings of the present study demonstrate that HL-RT, which was performed within the 70–90% 1RM range, generally yields higher muscle strength gains than LL-BFR, which was conducted within the 20–40% 1RM range. This observation is consistent with previous studies, emphasizing the superiority of high-load training for muscle strength development [17,18]. However, a study conducted by Liu et al. [27]. presented a contrasting view, indicating that LL-BFR can achieve comparable muscle strength gains to HL-RT. The discrepancies among studies might stem from multiple factors, with subject age being a prominent one. Meta-regression and subgroup analyses by Chang et al. [18]. further corroborate the aforementioned finding, indicating that older individuals may derive greater benefits from LL-BFR compared with younger individuals.

When implementing LL-BFR, applying an individualized cuff pressure prescription is more appropriate than a generalized occlusion pressure prescription. Individualized prescriptions account for equipment variations (e.g., different cuff widths), effectively overcoming limitations posed by such variability [28]. Further moderation factor analysis reveals that LL-BFR with individualized pressures can, in some instances, yield comparable muscle strength gains to HL-RT, differing from the findings of Lixandrão et al. [17], probably because of differences in subject characteristics and sample sizes. Notably, using a given absolute pressure (i.e., non-individualized) may result in varying degrees of vascular restriction because of differences in limb circumference, individual blood pressure, and cuff width, potentially compromising training effectiveness [29]. Therefore, individualized pressure prescriptions, made by adjusting the pressure to accommodate individual differences, ensure the efficacy and safety of training. Our results underscore the importance of adopting individualized pressure prescriptions in LL-BFR, providing a scientific basis for more precise and effective training plans.

Regarding the influence of cuff inflation modes on LL-BFR training outcomes, existing research presents mixed conclusions. Acute studies have found that continuous-pressure LL-BFR outperforms intermittent-pressure LL-BFR in muscle strength gains at 30% 1RM in female adolescents [30]. Conversely, a male-athlete-focused acute study revealed superior muscle strength gains with intermittent-pressure LL-BFR at the same load [31]. Such discrepancies may stem from gender differences, with our study partially supporting the findings of Li et al. [31]. In long-term interventions, a meta-analysis by Zhou et al. [32] suggests that intermittent cuff inflation modes outperform continuous inflation in enhancing aerobic capacity among healthy adults. Another meta-analysis on resistance training indicates that intermittent-pressure LL-BFR achieves comparable muscle strength gains to HL-RT, whereas continuous-pressure LL-BFR falls short [18]. These results are consistent with our conclusion, thereby supporting the superiority of intermittent-pressure LL-BFR in training outcomes.

When constructing exercise prescriptions, the integration of training frequency and duration is paramount. Prior research has shown that higher training frequencies and extended training periods are crucial for enhancing the effects of BFR training [33,34,35]. However, these studies have neglected the potential synergistic effects between frequency and duration. Building upon the median grouping strategies of previous studies [17], we applied this approach to categorize subjects based on the number of training sessions. Our results indicate that LL-BFR with a higher number of training sessions achieves comparable training effects to HL-RT, whereas with a lower number of training sessions, LL-BFR falls short. This finding underscores the need to consider the interplay between training frequency and duration when personalizing exercise prescriptions to maximize training outcomes.

### 4.2. Muscle Mass

Muscle Cross-Sectional Area is a direct measure of muscle mass increase. Similar to the analysis of muscle strength, the modulating effects on muscle mass were also examined. However, occlusion pressure prescriptions, cuff inflation patterns, and number of training sessions showed no significant effect on muscle mass improvement. This finding further supports our conclusion that LL-BFR and HL-RT have comparable muscle mass–enhancing effects, unperturbed by these modulating factors. Therefore, the two training modalities may share similar mechanisms and pathways in promoting muscle hypertrophy.

A distinctive feature of LL-BFR training lies in its ability to elevate metabolic stress during exercise through blood flow restriction. This metabolic stress can stimulate the release of muscle growth factors such as the insulin-like growth factor-1 and the modulation of myostatin, thereby fostering muscle protein synthesis [9]. By contrast, HL-RT directly elicits mechanical stress through high-load muscle contractions, which similarly activates these growth pathways [36]. Thus, despite their distinct initial stimuli, both methods may ultimately converge on comparable physiological responses.

Although this study did not uncover significant effects of occlusion pressure prescriptions, cuff inflation patterns, or number of training sessions on muscle mass gains, these factors remain crucial in training practice. They profoundly influence the overall training experience and recovery process for individuals. Different cuff inflation patterns and pressure settings can affect training comfort [30], pain perception [37], and potential injury risks [38]. The large-scale surveys conducted by Nakajima et al. [39] revealed the common, mild, and reversible side effects of BFR training, such as subcutaneous hematoma and transient numbness, emphasizing the importance of personalized adjustments for enhancing safety and compliance. Therefore, to enhance safety and subject compliance, intermittent and individualized pressurization schemes in LL-BFR are paramount. Intermittent cuff inflation effectively augments metabolic stress within muscles while shortening ischemic durations and mitigating associated discomfort, thereby optimizing the overall training outcomes [40]. This approach is particularly suitable for individuals with low tolerance to ischemic pain and discomfort, as it enhances their training adherence [41]. Meanwhile, individualized pressurization strategies ensure accurate cuff pressure application, maximizing the benefits for subjects while ensuring training safety and effectiveness. Thus, in practical applications, coaches and athletes should make precise adjustments based on individual conditions to ensure training safety and efficacy.

In conclusion, the findings of this study highlight the potential benefits of low-load resistance training combined with blood flow restriction LL-BFR in enhancing muscle strength and hypertrophy among untrained males. Notably, the application of individualized pressure prescriptions and intermittent cuff inflation modes significantly improved muscle strength gains in the LL-BFR group, making it a viable alternative to HL-RT. Therefore, in the practice of blood flow restriction training, we recommend the use of individualized pressure settings combined with intermittent cuff inflation to optimize training outcomes. This approach may have broad applications in sports performance enhancement, rehabilitation, and general health improvement by providing an effective and low-impact training method.

### 4.3. Limitations

First, the overall quality of studies included in this analysis was generally low because of the practical difficulties in fully blinding the subjects and experimenters in real-world settings. Second, despite efforts to control potential confounders across different populations and interventions, notable variations in the implementation of interventions (e.g., varying pressure levels and differences in training volumes) may still exist among studies. These variations could introduce bias during synthesis analysis, potentially affecting the stability of results and consistency of conclusions. Finally, the repeated citation of multiple related outcomes from the same study may have affected the homogeneity of results to a certain extent.

## 5. Conclusions

HL-RT outperforms LL-BFR in muscle strength gains. However, under appropriate conditions, such as individualized pressure settings, intermittent pressure application, and a high number of training sessions, LL-BFR can achieve comparable muscle strength gains to HL-RT, emphasizing the importance of personalized training programs. Their equivalence in muscle mass gains indicates that LL-BFR serves as an effective alternative for individuals who cannot undertake HL-RT because of physical limitations or injury concerns.

Additionally, in the practice of blood flow restriction training, we recommend combining individualized pressure settings with intermittent pressure application to optimize training outcomes and ensure safety and subject adherence.

## Data Availability

The original contributions presented in the study are included in the article/Supplementary Material, and further inquiries can be directed to the corresponding authors.

## Supplementary Materials

The following supporting information can be downloaded at: https://www.mdpi.com/article/10.3390/life14111442/s1, Table S1: Search strategy to identify the relevant articles on PubMed; Table S2: The basic characteristics of the included literature and the changes in the muscle strength indicators; Table S3: The basic characteristics of the included literature and the changes in the muscle mass indicators.
