# Supplementary material for: Effects of Blood Flow Restriction Training on Muscle Strength and Hypertrophy in Untrained Males: A Systematic Review and Meta-Analysis Based on a Comparison with High-Load Resistance Training
Source: Life (Basel). 2024 Nov 7;14(11):1442. doi: 10.3390/life14111442 (PMC11595635; doi:10.3390/life14111442)
Supplement: Supplementary file 1 [file life-14-01442-s001.zip › life-3247276-supplementary.pdf]

Effects of Blood Flow Restriction Training on Muscle Strength and Hypertrophy in Untrained Males: A Systematic Review and Meta-analysis Based on a Comparison with High-Load Resistance Training

Supplementary data

Supplementary Table S1. Search strategy to identify the relevant articles on PubMed.

**PubMed:**	("Blood Flow Restriction Therapy"[Mesh] OR BFR[Title/Abstract] OR Blood Flow Restriction[Title/Abstract] OR KAATSU[Title/Abstract] OR blood flow occlusion[Title/Abstract] OR Occlusion training[Title/Abstract] OR occluded blood flow[Title/Abstract] OR restricted blood flow[Title/Abstract] OR vascular restriction[Title/Abstract] OR vascular occlusion[Title/Abstract]) AND ("Muscle Strength"[Mesh] OR Arthrogenic Muscle Inhibitions[Title/Abstract] OR Inhibition, Arthrogenic Muscle[Title/Abstract] OR Muscle Inhibition, Arthrogenic[Title/Abstract] OR repetition maximum[Title/Abstract] OR 1RM[Title/Abstract] OR MVC[Title/Abstract] OR maximum voluntary contraction[Title/Abstract] OR MVIC[Title/Abstract] OR maximal voluntary isometric contraction[Title/Abstract] OR muscle force[Title/Abstract] OR skeletal muscle[Title/Abstract] OR muscle fiber[Title/Abstract] OR muscle power[Title/Abstract] OR strength[Title/Abstract] OR muscle mass [Title/Abstract] OR hypertrophy [Title/Abstract] OR muscle size [Title/Abstract] OR muscle thickness [Title/Abstract] OR cross sectional area [Title/Abstract]) AND (randomized controlled trial[Publication Type] OR random*[Title/Abstract] OR placebo[Title/Abstract] OR RCT[Title/Abstract] OR triple blind*[Title/Abstract] OR allocation[Title/Abstract] OR single blind[Title/Abstract] OR double blind[Title/Abstract])

We have followed the above search strategy to find the relevant articles from the PubMed database.

Table S2. The Basic Characteristics of the Included Literature and the Changes in the Muscle Strength Indicators

Study	Participant Characteristics		Intervention Characteristics		Weeks, (Sessions)	Strength Measurement	Outcomes	Intergroup Comparison
	Age	N	Load	Sets×Reps, Rest				
Bemben et al., 2022 [23]	21.3 ± 2.5<br>20.9 ± 2.9	LL-BFR, 12<br>HL-RT, 12	20% 1RM<br>70% 1RM	30,3×15, 60s<br>3×10, 60s	6 (18)	Dynamic knee extension<br>Dynamic knee flexion	LL-BFR: 21%<br>HL-RT: 25–32%	→
Centner et al., 2019 [12]	27.1 ± 4.7<br>26.1 ± 4.2	LL-BFR, 11<br>HL-RT, 14	20–35% 1RM<br>70–85% 1RM	30,3×15, 60s<br>3×6–12, 60s	14(42)	Isometric plantar flexion	LL-BFR: 10%<br>HL-RT: 14%	→
Centner et al.,2022 [24]	28.4 ± 4.9<br>27.6 ± 4.3	LL-BFR, 14<br>HL-RT, 15	20–35% 1RM<br>70–85% 1RM	30,3×15, 60s<br>3×6–12, 60s	14(42)	Dynamic leg press<br>Dynamic knee extension	LL-FR: 34–51%<br>HL-RT: 37–38%	→
Centner et al., 2023 [25]	28.4 ± 4.9<br>27.6 ± 4.3	LL-BFR, 14<br>HL-RT, 15	20–35% 1RM<br>70–85% 1RM	30,3×15, 60s<br>3×6–12, 60s	14(42)	Dynamic plantar flexors	LL-BFR: 43.6%<br>HL-RT: 43.5%	→
Horiuchi et al., 2023 [26]	22.0 ± 2.0<br>22.0± 2.0	LL-BFR, 12<br>HL-RT, 12	30% 1RM<br>75% 1RM	4×20, 30s<br>3×10, 120s	4(16)	Dynamic knee extension<br>Dynamic leg press	LL-BFR: 12–14%<br>HL-RT: 14–15%	→
Laswati et al., 2018 [21]	33.0 ± 3.1<br>33.33 ± 3.14	LL-BFR, 6<br>HL-RT, 6	30% 1RM<br>70% 1RM	30,3×15, 30s<br>3×12, 120s	5(10)	Isokinetic elbow flexion	LL-BFR: 48%<br>HL-RT: 27%	↑
Laurentino et al., 2022 [13]	20.0 ± 4.5<br>23.6 ± 6.0	LL-BFR, 10<br>HL-RT, 9	20% 1RM<br>80% 1RM	4×15, 60s<br>4×8–10, 90s	8(16)	Dynamic knee extension	LL-BFR: 40%<br>HL-RT: 36%	→
Lixandrão et al., 2015 [10]	27.9 ± 8.3<br>29.2 ± 9.9	LL-BFR, 43<br>HL-RT, 9	20–40% 1RM<br>80% 1RM	2-3×15, 60s<br>2-3×10, 60s	12(24)	Dynamic knee extension	LL-BFR: 10%–13%<br>HL-RT: 22%	↓
Martín-Hernández et al., 2013 [15]	20.7 ± 1.6<br>20.7 ± 2.3	LL-BFR, 20<br>HL-RT, 11	20% 1RM<br>85% 1RM	30,3×15, 60s<br>3×8, 60s	5(10)	Dynamic knee extension<br>Isokinetic knee extension	LL-BFR: 6–7%, 2–6%<br>HL-RT: 18%, 7–8%	↓
Ozaki et al., 2013 [20]	23.0 ± 0<br>24.0 ± 1.0	LL-BFR, 10<br>HL-RT, 9	30% 1RM<br>75% 1RM	30,3×15, 30s<br>3×10, 120–180s	6(18)	Dynamic bench press	LL-BFR: 9%<br>HL-RT: 18%	→
Ramis et al., 2020 [22]	23.52 ± 2.77<br>24.46 ± 2.56	LL-BFR, 15<br>HL-RT, 13	30% 1RM<br>80% 1RM	4×21-23, 120s<br>4×8, 120s	8(24)	Isokinetic elbow flexion<br>Isometric elbow flexion<br>Isokinetic knee extension<br>Isometric knee extension	LL-BFR: 10–11%, 6–9%<br>HL-RT: 11%–22%, 17%–18%	→
Sieljacks et al., 2019 [16]	23.7 ± 2.43	LL-BFR, 12<br>HL-RT, 12	30% 1RM<br>70% 1RM	4×failure, 30s<br>4×10–12, 180s	6(18)	Isometric knee extension,<br>Dynamic knee extension	LL-BFR: 6–10%<br>HL-RT: 13–23%	↓
Yasuda et al., 2011 [14]	23.4 ± 1.3<br>25.3 ± 2.9	LL-BFR, 10<br>HL-RT, 10	30% 1RM<br>75% 1RM	30,3×15, 30s<br>3×10, 120–180s	6(18)	Dynamic bench press<br>Isometric elbow extensors	LL-BFR: 9%, 0<br>HL-RT: 20%, 11%	↓

LL-BFR, Low load resistance training combined with blood flow restriction; HL-RT, High load resistance training; 1RM, One maximum repetition; 30, 3x15, 60s, 30 reps for the first set, 15 reps for sets 2-4, with a 60-second rest between sets; →, No significant between-group difference; ↓, Trend toward greater muscle strength gains for HL-RT; ↑, Trend toward greater muscle strength gains for LL-BFR

Table S3. The Basic Characteristics of the Included Literature and the Changes in the Muscle Mass Indicators

Study	Participant Characteristics		Intervention Characteristics		Weeks, (Sessions)	Mass Measurement	Outcomes	Intergroup Comparison
	Age	N	Load	Sets×Reps, Rest				
Bemben et al., 2022 [23]	21.3 ± 2.5	LL-BFR, 12	20% 1RM	30,3×15, 60s	6 (18)	Thigh-CSA	LL-BFR: 27%	→
	20.9 ± 2.9	HL-RT, 12	70% 1RM	3×10, 60s			HL-RT: 22%	
Centner et al., 2019 [12]	27.1 ± 4.7	LL-BFR, 11	20–35% 1RM	30,3×15, 60s	14(42)	Gastrocnemius medialis-CSA	LL-BFR: 9%	→
	26.1 ± 4.2	HL-RT, 14	70–85% 1RM	3×6–12, 60s			HL-RT: 8%	
Laurentino et al., 2022 [13]	20.0 ± 4.5	LL-BFR, 10	20% 1RM	4×15, 60s	8(16)	Quadriceps-CSA	LL-BFR: 6%	→
	23.6 ± 6.0	HL-RT, 9	80% 1RM	4×8–10, 90s			HL-RT: 6%	
Lixandrão et al., 2015 [10]	27.9 ± 8.3	LL-BFR, 43	20–40% 1RM	2-3×15, 60s	12(24)	Quadriceps-CSA	LL-BFR: 1%–5%	LL-BFR (40%1RM): →, LL-BFR (20%1RM): ↓
	29.2 ± 9.9	HL-RT, 9	80% 1RM	2-3×10, 60s			HL-RT: 6%	
Ozaki et al., 2013 [20]	23.0 ± 0	LL-BFR, 10	30% 1RM	30,3×15, 30s	6(18)	Triceps-CSA	LL-BFR: 7%	→
	24.0 ± 1.0	HL-RT, 9	75% 1RM	3×10, 120–180s		Pectoralis-CSA	HL-RT: 12%	
Ramis et al., 2020 [22]	23.52 ± 2.77	LL-BFR, 15	30% 1RM	4×21–23, 120s	8(24)	Biceps-MT	LL-BFR: 3–7%	→
	24.46 ± 2.56	HL-RT, 13	80% 1RM	4×8, 120s		Quadriceps-MT	HL-RT: 4–9%	
Yasuda et al., 2011 [14]	23.4 ± 1.3	LL-BFR, 10	30% 1RM	30,3×15, 30s	6(18)	Triceps-CSA	LL-BFR: 5–8%	→
	25.3 ± 2.9	HL-RT, 10	75% 1RM	3×10, 120–180s		Pectoralis-CSA	HL-RT: 9–18%	

LL-BFR, Low load resistance training combined with blood flow restriction; HL-RT, High load resistance training; 1RM, One maximum repetition; CSA, Cross sectional area; MT, Muscle thickness; 30, 3x15, 60s, 30 reps for the first set, 15 reps for sets 2-4, with a 60-second rest between sets; →, No significant between-group difference; ↓, Trend toward greater muscle mass gains for HL-RT; ↑, Trend toward greater muscle mass gains for LL-BFR
